# The Relation Between Post-Operative Surgical Site Infection and Time to Start Adjuvant Treatment in Ovarian and Uterine Cancers

**DOI:** 10.3390/curroncol32080474

**Published:** 2025-08-21

**Authors:** Karlijn M. C. Cornel, Julie My Van Nguyen, Lilian T. Gien, Allan Covens, Danielle Vicus

**Affiliations:** 1 Division of Gynecologic Oncology, University of Toronto, Toronto, ON M5G 1E2, Canada; karlijncornel@gmail.com (K.M.C.C.); lilian.gien@sunnybrook.ca (L.T.G.); al.covens@sunnybrook.ca (A.C.); 2 Department of Obstetrics and Gynecology, Division of Gynecologic Oncology, Juravinski Hospital and Cancer Centre, McMaster University, Hamilton, ON L8S 4L8, Canada; nguyenjmv@hhsc.ca; 3 Division of Gynecologic Oncology, Sunnybrook Health Sciences Centre, University of Toronto, Toronto, ON M4N 3M5, Canada

**Keywords:** uterine cancer, ovarian cancer, surgical site infection, oncologic outcomes

## Abstract

Surgical site infections (SSIs) occur in 10–15% of patients and are linked to up to 29% of delays in starting adjuvant treatment. This study assessed the association between SSIs in patients with ovarian and uterine cancer and their impact on time to adjuvant therapy and oncologic outcomes. Patients who underwent surgery from 1 January 2015 to 30 September 2017 were included, using institutional National Surgical Quality Improvement Program (NSQIP) data and chart reviews. Among 371 patients (median follow-up 4.1 years), 243 (65.5%) received adjuvant treatment. The median time to start was 39 days for chemotherapy, 61 days for radiotherapy, and 42 days for combined therapy (*p* < 0.001). Patients with ovarian cancer began treatment sooner than those with uterine cancer (39 vs. 52 days, *p* < 0.001), but no significant difference was observed between those with or without SSIs. In 238 patients with uterine cancer, those with SSIs had a twofold higher recurrence risk (HR 1.97, *p* = 0.022) and over threefold lower overall survival (HR 3.45, *p* = 0.018). Multivariable analysis showed that surgical route and disease stage were independent predictors; SSI was not an independent factor. No survival difference related to SSIs was found in patients with ovarian cancer. Further research is needed to clarify the impact of SSIs on treatment timing and recurrence.

## Simple Summary

Ovarian and uterine cancers are mainly treated with a combination of surgery followed by adjuvant chemotherapy treatment and/or radiation treatment. Post-surgical complications, including surgical site infection, might influence the time to start adjuvant treatment, with a possible impact on patients’ outcomes. In this study, we evaluated the time to start adjuvant treatment after surgery, comparing patients with a surgical site infection to those without. A statistically significant difference in time to start treatment was found between patients with ovarian and uterine cancer, as well as between the time to start chemotherapy treatment compared to radiation treatment. Although no difference was seen between patients with or without a surgical site infection. Patients with uterine cancer and a surgical site infections had a significantly higher rate of recurrent disease and a poorer overall survival compared to patients without a surgical site infection. No difference was seen for recurrence-free and overall survival in patients with ovarian cancer. Since surgery is a significant part of treatment in patients with gynecologic malignancies, it is crucial to further investigate the impact of surgical-related complications on adjuvant treatment and oncologic outcomes. With the goal of improving and evolving oncological care for patients with gynecologic malignancies.

## 1. Introduction

Gynecologic cancers are common and account for 115,130 newly diagnosed cases each year and up to 32,830 cancer-related deaths in North America [1]. The management of gynecologic malignancies predominantly relies on a multimodality approach involving surgical intervention along with adjuvant modalities such as chemotherapy (CT), radiotherapy (RT), or a combination of both (CRT). This strategy aims to enhance both disease-free survival and overall survival. The interval between surgery and the start of adjuvant treatment can be prolonged due to postoperative complications, potentially impacting oncologic outcomes.

Manher et al. showed a decrease in disease-free and overall survival for every extra week between surgery and the start of adjuvant treatment in a population with advanced ovarian cancer [2]. These results have been supported by others who have found a link between the delayed start of adjuvant CT and prognosis [2,3,4]. However, other studies have not demonstrated a significant difference between the time to start adjuvant treatment and oncologic outcome [5,6,7]. The ideal time between surgery and adjuvant CT is not entirely clear; commonly, a maximum cut-off of 6 weeks (42 days) is used, although some advocate initiating adjuvant CT within 21 days of surgery [2,4,8,9].

Surgical site infection (SSI) is a common post-surgical complication that can cause a delay in the start of adjuvant treatment. SSIs are common after surgery for gynecologic malignancies and occur in 10–15% of cases [3]. They often result in a prolonged hospital stay, and patients with an SSI have a higher chance of readmission and present more often to the emergency department [10,11]. O’Donnell et al. showed that 29% of patients undergoing surgical procedures for gynecologic malignancies and subsequently developing SSIs experienced delays or cancellations in the commencement of adjuvant treatment [12,13].

Our group previously showed that BMI > 30 kg/m^2^, smoking, and surgery by laparotomy are independent risk factors for the development of SSIs [14]. Other risk factors reported in the literature include hyperglycemia, albumin, surgical complexity, and residual/increased disease dissemination [3,15,16]. It has been shown that the chances of developing an SSI can be reduced by using specific perioperative measures [14,17,18]. We successfully reduced the number of SSIs at our institution by 55% by implementing a peri-operative bundle [14].

The primary aim of this study was to evaluate the association between surgical site infections (SSIs), including superficial, deep, and organ-space infections (as defined by the NSQIP), and time to start adjuvant treatment. Secondary objectives included identifying risk factors for SSI, assessing the association between SSIs and time to recurrence, as well as determining overall survival in patients undergoing surgery for an ovarian or uterine cancer.

## 2. Methods

### 2.1. Study Design and Population

This retrospective cohort study included all patients who underwent elective surgery for an ovarian or uterine cancer, confirmed on final pathology, between 1 January 2016 and 30 September 2017 in a tertiary center, captured in the institutional National Surgical Quality Improvement Program (NSQIP) database [14]. The NSQIP database is a prospectively collected, validated, risk-adjusted, outcomes-based program designed to measure and improve the quality of surgical care [19].

Patient demographics, preoperative comorbidities, oncologic details, intraoperative factors, and 30-day outcomes and complications were previously collected using NSQIP institutional data and chart review [14]. We further collected the time to initiation of adjuvant therapy in days and the time to recurrence (both calculated from the date of surgery) in days, along with overall survival in months. Patients were stratified by type of adjuvant treatment (chemotherapy, radiotherapy, or a combination of chemotherapy and radiotherapy) and type of cancer (ovarian cancer or uterine cancer). Listwise deletion (complete case analysis) was used in cases of missing data.

### 2.2. Definitions

SSIs (superficial, deep, and organ-space) were defined according to the Centers for Disease Control and Prevention criteria [20,21] and were identified through the institutional NSQIP database. The number of overall SSIs was defined as the number of episodes during which patients incurred one or more types of SSI within 30 days of surgery. For example, if a patient concurrently had an organ-space SSI and a superficial SSI, this was categorized as one overall SSI episode.

### 2.3. Statistical Analysis

All statistical analyses were performed using IBM SPSS Statistics version 29.0.

Descriptive statistics were used, continuous variables were compared using the Mann–Whitney U test, and categorical data were compared using the Chi-squared test.

Univariate logistic regression was used to identify risk factors for SSI, both in the overall population as well as per subgroup of patients with ovarian cancer and uterine cancer. Factors found to be statistically significant and clinically meaningful were included in the multivariate logistic regression model.

The Kaplan–Meier and log-rank methods were used for recurrence-free survival and overall survival. Univariate Cox proportional hazards analysis was used to evaluate the magnitude of significance when the log-rank test was significant. A *p*-value of <0.05 was considered statistically significant.

## 3. Results

### 3.1. Overall Population

A total of 563 patients with a (suspected or confirmed) gynecologic malignancy who underwent surgery were prospectively included between 1 January 2016 and 30 September 2017 at a tertiary university hospital [14].

Out of this dataset, 371 patients were included in the analysis; 20 patients (3.6%) were lost to follow up, 42 patients (7.5%) received their adjuvant treatment in other medical centers, 69 patients (12.3%) were excluded due to a benign or pre-malignant pathology on the final specimen, and 8 patients (1.4%) were excluded due to a non-gynecological malignancy on final pathology. Another 43 patients (7.6%) were excluded from this analysis because they were diagnosed with a gynecologic cancer other than a uterine or ovarian cancer, and 10 patients (1.8%) were excluded because they were treated with hormonal treatment.

Of the 371 patients analyzed, the median follow-up time was 4.1 years (range: 0.1–7.6 years). Patient characteristics are shown in Table 1. Median BMI (kg/m^2^) was 27.9 (range: 14.8–51.9). Only 17 patients (4.6%) had a history of smoking. Diabetes mellitus was seen in 57 patients (15.4%). In total, 133 patients (35.8%) were treated for ovarian cancer, and 238 (64.2%) patients were treated for uterine cancer (Table 1). For details on histological type and International Federation of Gynecology and Obstetrics (FIGO) stage, see Table S1.

A total of 243 patients (65.5%) received adjuvant treatment: 128 (52.7%) received chemotherapy (CT), 71 (29.2%) received radiotherapy (RT), and 44 (18.1%) received a combination of chemotherapy and radiotherapy (CRT) (Table 2).

The median time from surgery to the start of adjuvant treatment was 44 days (range: 13–160 days) and was statistically significantly different (*p* < 0.001) between the types of adjuvant treatment. The median time from surgery to the start of treatment was 39 days (range: 17–93 days) for chemotherapy treatment, 64 days (range: 38–126 days) for radiotherapy treatment, and 42 days (range: 13–160 days) for chemotherapy and radiotherapy treatment (Table 2).

In total, there were 38 patients (10.2%) who developed an SSI after surgery. On univariate analysis, patients with an SSI had a statistically significantly higher BMI, were more likely to be smokers, were more likely to have a procedure through laparotomy, had higher (median) blood loss, had a higher percentage of intra-operative complications, had a longer hospital stay, and had an increased number of reoperations or readmissions within 30 days (Table 1).

Multivariate analysis for SSI was statistically significant for BMI > 30 kg/m^2^, smoking, and route of surgery (protective effect of laparoscopy versus laparotomy), with odds ratios (ORs) of 4.5 (95% confidence interval (CI) 2.1–10.0), 6.5 (95% CI 1.9–22), and 0.2 (95% CI 0.08–0.6); see Table 3. Although reoperation < 30 days and readmission < 30 days were statistically significant on univariate analysis, we did not include these in the multivariate analysis due to the number of events (38 patients with an SSI out of a total of 371), as well as the greater likelihood that those differences might be caused by an SSI rather than related to the development of an SSI.

### 3.2. SSI and Adjuvant Treatment

The overall median time to start adjuvant treatment was 45 days for patients without an SSI compared to 42 days for patients with a post-operative SSI, which was not statistically significantly different (*p* = 0.441). However, there was a statistically significant difference between the types of adjuvant treatment (chemotherapy versus radiotherapy versus chemotherapy plus radiotherapy), with *p* ≤ 0.001. Analysis comparing patients with and without an SSI by subgroup of adjuvant treatment (chemotherapy versus radiotherapy versus chemotherapy plus radiotherapy) did not show statistically significant differences (*p* ≥ 0.05, Table 3). The median time to adjuvant treatment was 79.5 days in those with an SSI versus 61 days in those without an SSI for radiotherapy (*p* = 0.547), 42 days versus 39 days for chemotherapy (*p* = 0.383), and 42.0 days in both patients with and without SSI for chemotherapy and radiotherapy (*p* = 0.914).

However, there was a statistically significant difference in the time to start adjuvant treatment for patients diagnosed with ovarian cancer: 39 days compared to 52 days for patients with uterine cancer (*p* ≤ 0.001) (Table 3).

### 3.3. Impact of SSI on Recurrence and Overall Survival

There were 148 (39.9%) patients who developed disease recurrence: 128 (38.4%) in the group without an SSI and 20 (52.6%) in the group with an SSI (Table 4). A total of 69 (51.9%) and 79 (33.2%) of the patients treated for ovarian and uterine cancer, respectively, developed recurrent disease (Table 4).

**Figure 1:**
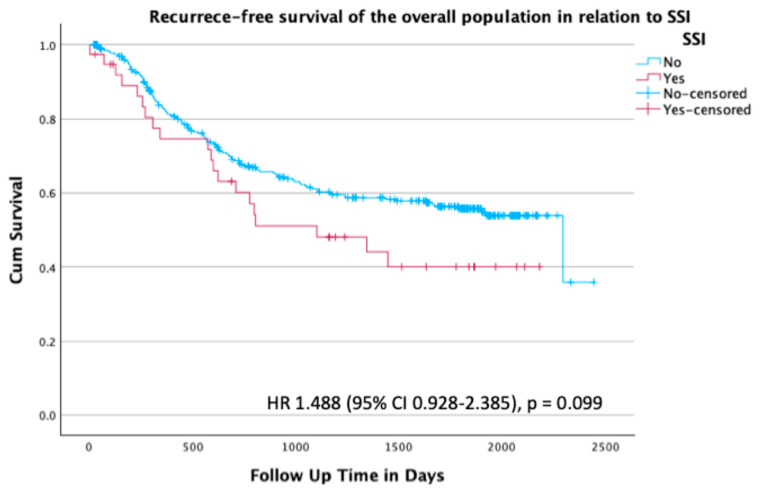
Kaplan–Meier survival plot for recurrence-free survival of the overall population stratified by presence or absence of SSI; hazard ratio (HR) 1.488 (95% confidence interval (CI) 0.928–2.385), *p* = 0.099.

In the overall population, there was no statistical difference in recurrence-free survival between patients with and without an SSI (*p* = 0.110, HR 1.49 (95% CI 0.928–2.385)) (Figure 1).

**Figure 2:**
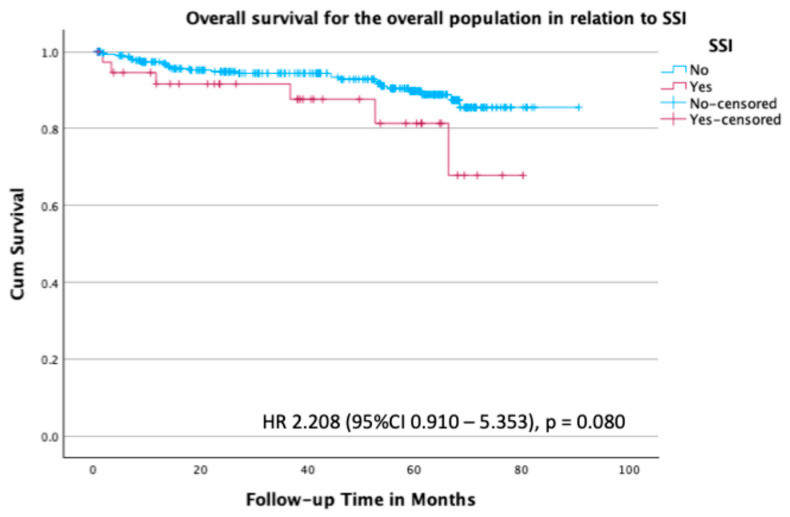
Kaplan–Meier survival plot for overall survival in the overall population stratified by the presence or absence of SSI, with an HR of 2.208 (95% CI 0.910–5.353; *p* = 0.080).

Overall survival in the total population in relation to SSI was not statistically significant, with a hazard ratio (HR) of 2.21 (95% CI 0.910–5.353; *p* = 0.080) (Figure 2).

### 3.4. SSI and Disease Recurrence in Patients with Uterine Cancer

In this section, we discuss the results for the subgroup of patients with uterine cancer. SSI was observed in 26 out of 238 patients (10.7%); 22 out of 94 (23.4%) patients underwent laparotomy, and 4 out of 148 (2.7%) were treated by laparoscopy.

In the univariate analysis assessing risk factors for the development of SSI in patients with uterine cancer, a statistically significant difference in baseline characteristics was found for BMI, smoking, route of surgery, stage of disease, estimated blood loss, and intraoperative complications (Table S2).

In relation to post-surgical characteristics, a significant difference was observed in the median length of hospital stay and the percentage of patients who underwent reoperation or were readmitted within 30 days, at 7.7% versus 0.9% and 19.2% versus 2.8%, respectively, for patients with an SSI versus those without an SSI (Table S2). On multivariate analysis BMI > 30 kg/m^2^, smoking and route of surgery (laparoscopy versus laparotomy) are independently related to the development of SSI in patients with uterine cancer, with ORs of 3.0 (95% CI 1.1–8.4), 7.5 (95% CI 1.4–39.4), and 0.13 (95% CI 0.7–4.4), respectively (Table S3).

**Figure 3:**
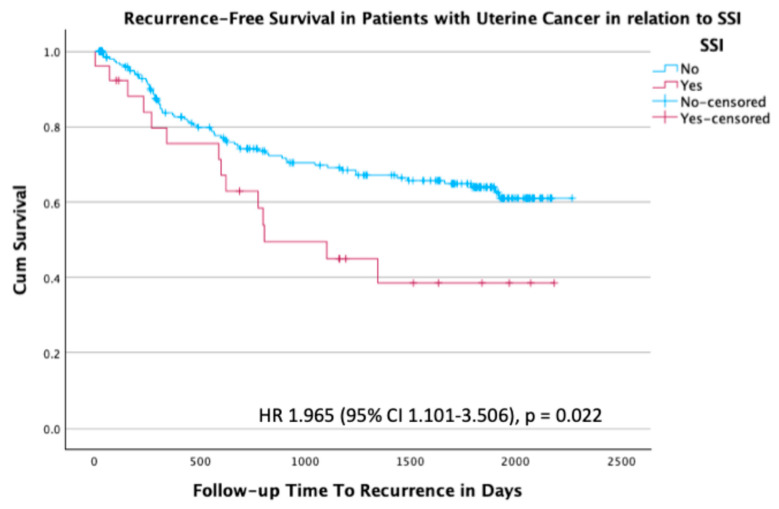
Kaplan–Meier survival plot for recurrence-free survival in the subgroup of patients with uterine cancer stratified by the presence or absence of SSI, with an HR of 1.965 (95% CI 1.101–3.506; *p* = 0.022).

At the time of last follow-up, 159/238 patients (66.8%) were alive without disease, 60/238 patients (25.2%) were alive with disease, and 19/238 patients (8.0%) had died (Table 5). A total of 79 patients (33.2%) with uterine cancer had recurrence. There was an almost twofold increased rate of recurrence in patients with an SSI compared to those without an SSI, with an HR of 1.96 (95% CI 1.101–3.506; *p* = 0.022) (Figure 3).

**Figure 4:**
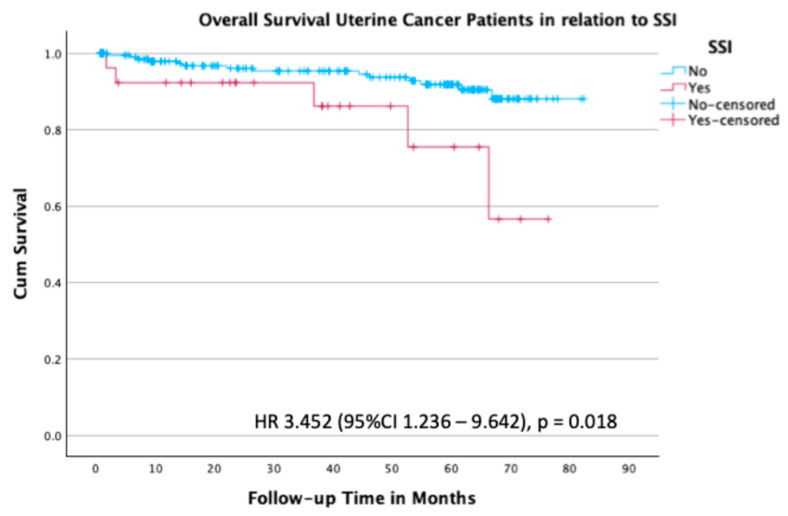
Kaplan–Meier survival plot for overall survival in the subgroup of patients with uterine cancer stratified by the presence or absence of SSI.

Overall survival results for patients with uterine cancer are shown in Figure 4. Patients with uterine cancer who had an SSI had a 3.5-fold increased risk of death in comparison to those without an SSI, with an HR of 3.45 (95% CI 1.236–9.642; *p* = 0.018) (Figure 4).

### 3.5. SSI and Disease Recurrence in Patients with Ovarian Cancer

In this section, we discuss the results for the subgroup of patients with ovarian cancer. Univariate analysis of risk factors for SSI in patients diagnosed and treated for ovarian cancer did not show any significant differences between the groups with and without an SSI (Table S4). However, there was a statistically significant difference in post-surgical variables, including reoperation and readmission within 30 days of surgery, with rates of 2.3% versus 0.8% and 41.7% versus 6.0%, respectively, for patients with an SSI versus patients without an SSI (Table S4). Since there were no significant differences in overall baseline characteristics, a multivariate analysis was not performed.

At the time of last follow-up, 68/133 patients (51.1%) were alive without disease, 51/133 patients (38.3%) were alive with disease, and 14/133 patients (10.5%) had died (Table 5). A total of 65 patients (48.8%) with ovarian cancer developed recurrent disease.

Kaplan–Meier analysis did not show a statistically significant difference in recurrence-free survival in the group of patients treated for ovarian cancer with or without an SSI (HR 0.98; 95% CI 0.424–2.267; *p* = 0.962) (Figure S1).

**Figure 5:**
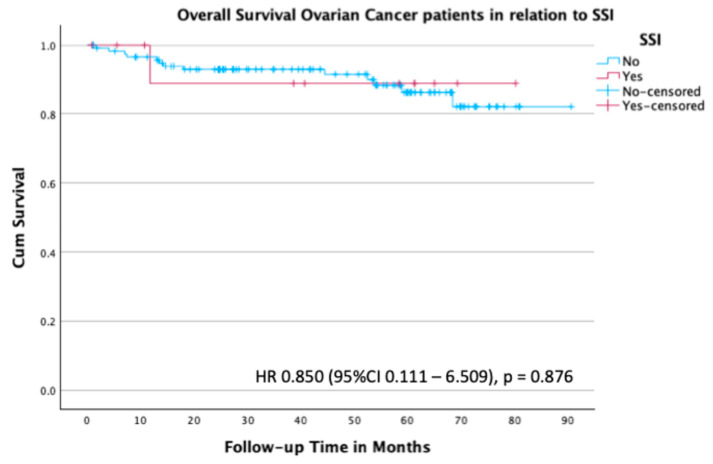
Kaplan–Meier survival plot for overall survival in the subgroup of patients with ovarian cancer stratified by the presence or absence of SSI.

In line with these findings, the Kaplan–Meier analysis for overall survival did not show a significant difference between patients with and without an SSI (HR 0.85; 95% CI 0.111–6.509; *p* = 0.876) (Figure 5).

## 4. Discussion

SSI is one of the most common postoperative complications, and there are limited data on its impact on the time to start adjuvant treatment and its effect on survival in patients treated for gynecological malignancies. In our cohort of 371 patients with ovarian or uterine cancer, a total of 8.7% were diagnosed with an SSI; this percentage is in line with previous literature [15]. Although ovarian and uterine cancer have different tumor biology and adjuvant treatment options, the majority of surgical procedures in gynecologic oncology practice are similar; therefore, we analyzed our population as a whole and performed subgroup analyses for patients with ovarian cancer and those with uterine cancer.

In our study, we found that patients with surgical site infections (SSIs) had a longer median time between surgery and the start of chemotherapy (39 vs. 42 days; *p* = 0.383) or radiotherapy (61 vs. 79.5 days; *p* = 0.547) compared to those without an SSI. Furthermore, in our population, we also demonstrated that SSIs had a negative impact on both the length of hospital stay and the 30-day readmission rate. The relationship between SSIs and prolonged hospital stay has been described previously; however, causality has never been proven. Both prolonged stay due to SSI [12,14,22] and longer hospital stay have been reported as factors that increase the risk of developing an SSI [23]. However, these differences were not found to be statistically significant. Interestingly, we found a significant difference in the time to start of adjuvant treatment by diagnosis; those with ovarian cancer started treatment earlier than those with uterine cancer (39.0 versus 52.0 days, *p* ≤ 0.001). No difference in time to start of adjuvant therapy was seen in patients with or without an SSI within each disease site. This can be explained by the fact that the primary treatment for ovarian cancer is adjuvant chemotherapy, which can be started fairly soon after surgery, while at the time of inclusion, the primary adjuvant treatment for uterine cancer was radiotherapy or a combination of radiotherapy with chemotherapy for advanced disease, which may require additional time for planning and scheduling [24].

In our ovarian cancer population, the median time to start chemotherapy was 39 days. This is in line with most guidelines, which recommend starting chemotherapy treatment within 6 weeks (42 days) of surgery [2,4,8,9]. In their meta-analysis on time to start adjuvant treatment in patients with ovarian cancer, which included 12 studies, Uson et al. showed that there was no significant difference in disease recurrence or death when comparing an ‘early start’ of adjuvant chemotherapy with a ‘late start’ of adjuvant chemotherapy [7] Interestingly, most of the articles included in the analysis compared ‘early start’, defined as starting treatment within 20 days after surgery, with ‘late start’, which included a start of chemotherapy within the recommended time frame of 30–42 days [7]. These results have been confirmed by Gadducci et al., who stratified the time to start chemotherapy treatment in advanced-stage ovarian cancer in four quartiles (starting within 11 days, within 21 days, within 31 days, and >32 days after surgery) and did not show any significant difference in response to chemotherapy treatment or overall survival [25]. Even though the range of the start of adjuvant chemotherapy treatment in our population diagnosed with ovarian cancer was wide (17–93 days), no significant difference was seen in recurrence-free survival, which is in line with the literature.

The median time to start radiotherapy treatment in our cohort was 61.0 days (range: 38–126 days). In previous large randomized controlled trials, adjuvant radiotherapy treatment for uterine cancers has been reported to be initiated within 8 weeks (56 days) of surgery [26,27,28,29]. However, Ghanem et al. did not show a significant effect on overall survival comparing starting RT < 8 weeks compared to starting RT ≥ 8 weeks in a large population of patients with early-stage uterine cancer [30]. This is in line with large randomized controlled trials, in which adjuvant radiotherapy treatment has been shown to reduce local recurrence rates without a significant effect on overall survival [27,29].

For the patients in the combination therapy group (chemotherapy plus radiotherapy), the median time to start treatment was closer to that of the group who received chemotherapy alone, with a median of 42.0 days. This can be explained by the fact that most patients begin with chemotherapy treatment, whereas radiotherapy treatment is given either after three cycles of chemotherapy and is followed by another three cycles of chemotherapy, or after completion of six cycles of chemotherapy. A small number of patients have been treated as per the PORTEC 3 protocol, starting with chemoradiation followed by four cycles of chemotherapy treatment. Patients treated for uterine cancer also did not show any significant difference in the start time of their adjuvant treatment when comparing patients with and without an SSI (median days 42.0 versus 42.0, *p* = 0.914).

Of the 239 patients with uterine cancer in our cohort, 79 (33.2%) recurred. There was an almost 2-fold increased rate of recurrence seen in patients with an SSI in comparison to those without an SSI (*p* = 0.030, HR 1.89). SSI negatively impacted overall survival, with an HR of 3.45 (*p* = 0.018). At the time of this study, the primary adjuvant treatment for early-stage endometrioid uterine cancer was adjuvant radiation treatment, as treatment based on molecular classifications (including p53 mutations) and immunotherapy in MMR-deficient tumors was not yet introduced [24,31,32]. The significant difference in our population is especially surprising, as radiation treatment for uterine cancer has not been shown to impact overall survival in large randomized controlled trials [26,28].

Univariate analysis in the population with uterine cancer identified multiple patient and surgical factors related to the risk of developing an SSI, including BMI, smoking, laparotomy, estimated blood loss during surgery, and ASA level. On multivariable analysis, BMI > 30 kg/m^2^, smoking, and route of surgery, with a protective effect of laparoscopy, were independently related to the development of SSIs. These results are in line with the existing literature on risk factors related to surgical site infections. We did not find any difference in recurrence rate in the group of patients treated for ovarian cancer when comparing patients with and without an SSI. Previously noted factors that have been shown to impact prognosis include microscopic resection and response to chemotherapy treatment; based on our results, these appear to be more important than the time to start chemotherapy treatment. There was no statistically significant difference in overall survival in the group of patients with ovarian cancer with or without an SSI in our study. In the literature, the relationship between SSI and overall survival is inconsistent. Tran et al. found a decrease in overall survival for patients with ovarian cancer who underwent primary cytoreductive surgery [3]. This was also supported by M. Szymankiewicz et al.; however, due to the retrospective nature of this study, there is a high risk of selection bias [6]. Unfortunately, many studies focusing on surgical site infections do not include an adequate follow-up period to report on oncological outcomes, which may influence the results published.

The strengths of our study include its use of a large cohort of patients, in which data were collected prospectively through the NSQIP to evaluate the implementation of an SSI prevention bundle. Furthermore, in this database, there is a clear definition of SSI, which prevents clinical bias. All patients underwent surgery for gynecologic malignancies, and due to the nature of the initial study, SSI data were closely monitored, resulting in well-documented rates. For this retrospective cohort, we included all patients with pathology-proven ovarian and uterine cancer. As the initial data were prospectively collected, our inclusion bias was limited. Utilizing NSQIP data ensured accurate data capture and high accuracy, limiting the chance of collection reporting bias. With a median follow-up period of 4.1 years, we ensured that we could account for early and late recurrences.

One of the main limitations is that we included patients with two different types of gynecological cancers, resulting in a heterogeneous group requiring different types of adjuvant treatment. However, this represents the majority of patients in the current gynecologic oncology practice and is therefore generalizable to other hospitals. Also, despite the large population, the number of SSIs was still relatively low. This might under-represent the impact of SSIs on patient outcomes. Furthermore, due to the limited number of events and the lack of factors related to disease progression and overall survival, we elected not to perform a multivariate Cox proportional hazard regression analysis, as the risk of bias was high. Moreover, other factors influence the risk of SSI, for example, laparotomy versus laparoscopy, BMI, intra-operative complications, and blood loss during surgery. In our population, the distribution between laparotomy and laparoscopy was unequally distributed, with more laparotomy procedures in the ovarian cancer population and more laparoscopy procedures in the uterine cancer population. Future research is needed to investigate additional prognostic factors and their impact on the development of distinct types of SSIs (deep SSI versus superficial SSI).

## 5. Conclusions

SSI may impact the time to adjuvant CT or RT; however, this was not found to be statistically significant in our study, possibly due to the limited number of events or a lack of effect. Although patients with uterine cancer with an SSI had an increased recurrence rate, further research is needed to clarify the impact of SSIs on time to adjuvant treatment, recurrence-free survival, and overall survival. Implementing appropriate measures to minimize the risk of SSI in patients with gynecologic cancers is recommended to optimize patient care and outcomes.

## Figures and Tables

**Table 1:** Descriptive statistics of patients with and without SSI.

	Total	Patients Without SSI	Patients with SSI	*p*-Value
**Number of patients**	371 (100%)	333 (89.8%)	38 (10.2)	
**Median BMI (kg/m^2^) (range)**	27.9 (14.8–51.9)	27.3 (14.8–50.8)	32.9 (18.4–51.9)	0.002
**Diabetes mellitus**				0.691
Yes	57 (15.4%)	52 (15.6%)	5 (13.2%)	
No	314 (84.6%)	281 (84.4%)	33 (86.8%)	
**Smoking**				0.004
Yes	17 (4.6%)	11 (3.3%)	6 (15.8%)	
No	354 (95.4%)	322 (96.7%)	32 (84.2%)	
**Median follow-up years (range)**	4.1 (0.1–7.6)	4.3 (0.1–7.6)	3.3 (0.1–6.7)	0.238
**Type of gynecological cancer**				0.562
Ovarian	133 (35.8%)	121 (36.3%)	12 (31.6%)	
Uterine	238(64.2%)	212 (63.7%)	26 (68.4%)	
**Grade**				0.089
1	31 (8.4%)	30 (9.0%)	1 (2.6%)	
2	74 (19.9%)	70 (21.0%)	4 (10.5%)	
3	263 (70.9%)	230 (69.1%)	33 (86.9%)	
Unknown	3 (0.8%)	3 (0.9%)	0 (0%)	
**Lymph vascular space invasion**				0.923
Yes	159 (43.0%)	142 (42.8%)	17 (44.7%)	
No	157 (42.4%)	142 (2.8%)	15 (39.5%)	
Unknown	54 (14.6%)	48 (14.5%)	6 (15.8%)	
**Route of surgery**				<0.001
Laparotomy	213 (57.4%)	180 (54.1%)	33 (86.8%)	
Laparoscopy	158 (42.6%)	153 (45.9%)	5 (13.2%)	
**Median estimated blood loss (mL)**	200 (50–11,000)	200 (50–11,000)	500 (50–4500)	0.007
**ASA**				0.001
1	9 (2.4%)	8 (2.4%)	1 (2.6%)	
2	68 (18.3%)	63 (18.9%)	5 (13.2%)	
3	245 (66.0%)	225 (67.6%)	20 (52.6%)	
4	48 (12.9%)	37 (11.1%)	11 (28.9%)	
5	1 (0.3%)	0	1 (2.6%)	
**Bowel resection**				0.080
Yes	46 (12.4%)	38 (11.4%)	8 (21.1%)	
No	325 (87.6%)	295 (88.6%)	30 (78.9%)	
**Intraoperative complications**				0.003
Yes	21 (5.7%)	14 (4.2%)	7 (18.4%)	
No	350 (94.3%)	319 (95.8%)	31 (81.6%)	
**Median Length of hospital stay (days)**	3.0 (0–101)	3.0 (0–17)	5.0 (0–101)	<0.001
**Reoperation < 30 days**				<0.001
Yes	7 (1.9%)	2 (0.6%)	5 (13.2%)	
No	364 (98.1%)	331 (99.4%)	33 (86.8%)	
**Readmission < 30 days**				<0.001
Yes	24 (6.5%)	14 (4.2%)	10 (26.3%)	
No	347 (93.5%)	319 (95.8)%	28 (73.7%)	

**Table 2:** Univariate analysis comparing types of adjuvant treatment after surgery and time to treatment in patients with and without SSI.

	Total	Patients Without SSI	Patients with SSI	*p*-Value
**Adjuvant treatment**				0.447
Yes	243 (65.5%)	216 (64.9%)	27 (71.1%)	
No	128 (34.5%)	117 (35.1%)	11 (28.9%)	
**Type of adjuvant treatment**				0.193
Chemotherapy	128 (52.7%)	110 (50.9%)	18 (66.7%)	
Radiotherapy	71 (29.2%)	67 (31.0%)	4 (14.8%)	
Chemotherapy + radiotherapy	44 (18.1%)	39 (18.1%)	5 (18.5%)	
**Median time to adjuvant treatment (days)**	44.0 (13–160)	45.0 (13–160)	42.0 (27–158)	0.441
**Median time to adjuvant treatment per therapy type (days)**				<0.001
Chemotherapy	39.0 (17–93)	39.0 (17–72)	42.0 (27–93)	0.383
Radiotherapy	61.0 (38–126)	61.0 (38–126)	79.5 (48–125)	0.547
Chemotherapy + radiotherapy	42.0 (13–160)	42.0 (13–160)	42.0 (31–158)	0.914
**Median time to adjuvant treatment per primary malignancy (days)**				<0.001
Ovarian	39.0 (17–72)	39.0 (17–72)	36.5 (27–56)	0.843
Uterine	52.0 (13–160)	52.0 (13–160)	48.0 (31–158)	0.832

**Table 3:** Multivariate logistic regression analysis to determine independent risk factors for SSI in the overall population.

	*p*-Value	Odds Ratio	95% CI
**BMI < 30 kg/m^2^ vs. >30/kg/m^2^**	<0.001	4.544	2.065–9.996
**Smoking**	0.003	6.513	1.932–21.956
**Route of surgery (laparoscopy vs. laparotomy)**	0.003	0.211	0.077–0.581
**Bowel resection**	0.209	1.861	0.706–4.906

**Table 4:** Disease recurrence.

Recurrence	Total	Patients Without SSI	Patients with SSI	*p*-Value
Yes	153 (40.3%)	133(38.9%)	20 (52.6%)	0.091
No	227 (59.7%)	209(61.1%)	18 (47.4%)	
**Median time to recurrence (days)**	449.5 (38–1920)	447.0 (38–1920)	581.5 (70–1446)	0.810
**Number of recurrences per primary tumor**				
**Ovarian**				0.891
Yes	69 (51.9%)	63 (52.1%)	6 (50.0%)	
No	64 (48.1%)	58 (47.9%)	6 (50.0%)	
**Uterine**				0.018
Yes	79 (33.2%)	65 (30.7%)	14 (53.8%)	
No	159 (66.8%)	147 (69.3%)	12 (46.2%)	

**Table 5:** Outcomes in patients with ovarian and uterine cancer.

	Total	Patients Without SSI	Patients with SSI	*p*-Value
**Ovarian Cancer**				0.868
Alive without disease	68 (51.1%)	61 (50.4%)	7 (58.3%)	
Alive with disease	51 (38.3%)	47 (38.8%)	4 (33.3%)	
Death	14 (10.5%)	13 (10.7%)	1 9(8.3%)	
**Uterine Cancer**				0.039
Alive without disease	159 (66.8%)	147 (69.3%)	12 (66.8%)	
Alive with disease	60 (25.2%)	51 (34.6%)	9 (34.6%)	
Death	19 (8.0%)	14 (6.6%)	5 (19.2%)	

## Data Availability

The data shown in this study are available from the corresponding author upon reasonable request.

## Supplementary Materials

The following supporting information can be downloaded at: https://www.mdpi.com/article/10.3390/curroncol32080474/s1, Table S1: Descriptive statistics regarding histological type and FIGO stage for patients with and without SSI, Table S2: Univariate analysis for SSI in patients with uterine cancer, Table S3: Multivariate logistic regression for risk factors for SSI in patients with uterine cancer, Table S4: Univariate analysis for SSI in patients with ovarian cancer, Figure S1: Overall recurrence-free survival for patients with ovarian cancer stratified by SSI.
