# Supplementary material for: The Relation Between Post-Operative Surgical Site Infection and Time to Start Adjuvant Treatment in Ovarian and Uterine Cancers
Source: Curr Oncol. 2025 Aug 21;32(8):474. doi: 10.3390/curroncol32080474 (PMC12385515; doi:10.3390/curroncol32080474)
Supplement: Supplementary file 1 [file curroncol-32-00474-s001.zip › curroncol-3735245-supplementary.pdf]

Supplementary Tables and Figures.

Table S1. Descriptive statistics regarding histological type and FIGO stage for patients with and without SSI.

	Total	Patients without SSI	Patients with SSI
**Type of gynaecological cancer**			
**Ovarian**	133 (35.8%)	121 (36.3%)	12 (31.6%)
Serous	83 (62.4%)	74 (61.2%)	9 (75.0%)
Clear cell	14 (10.5%)	12 (9.9%)	2 (16.7%)
Endometrioid	18 (13.5%)	17 (14.0%)	1 (8.3%)
Low grade	4 (3.0%)	4 (3.3%)	0 (0%)
Other	10 (10.6%)	10 (11.6%)	0 (0%)
**Uterine**	238(64.2%)	212 (63.7%)	26 (68.4%)
Serous	59 (24.8%)	53 (25.0%)	6 (23.1%)
Endometrioid	128 (53.8%)	114 (53.8%)	14 (53.8%)
Clear cell	10 (4.2%)	10 (4.7%)	0 (0%)
Carcinosarcoma	23 (9.7%)	21 (9.9%)	2 (7.7%)
Sarcoma	12 (5.0%)	9 (4.2%)	3 (11.5%)
Other	6 (2.5%)	5 (2.4%)	1 (3.9%)
**FIGO stage**			
**Ovarian**			
IA	25 (18.8%)	25 (19.8%)	0 (0%)
IB	2 (1.5%)	2 (1.6%)	0 (0%)
IC	9 (6.7%)	7 (5.8%)	2 (16.7%)
II	7 (5.3%)	7 (5.8%)	0 (0%)
IIIA	10 (7.5%)	9 (7.4%)	1 (8.3%)
IIIB	5 (3.8%)	4 (3.3%)	1 (8.3%)
IIIC	64 (48.1)	56 (46.2%)	8 (66.7%)
IV	11 (8.3%)	11 (9.1%)	0 (0%)
**Uterine**			
IA	106 (44.5%)	98 (46.2%)	8 (30.8%)
IB	34 (14.7%)	32 (15.1%)	2 (7.7%)
II	21 (8.8%)	19 (9.0%)	2 (7.7%)
IIIA	9 (3.8%)	8 (3.8%)	1 (3.8%)
IIIB	2 (0.8%)	2 (0.9%)	0 (0%)
IIIC	40 (16.8%)	34 (16.0%)	6 (23.1%)
IV	26 (10.9%)	19 (9.0%)	7 (26.9%)

Table S2. Univariate analysis of risk factors for SSI in patients with uterine cancers.

	Total (n=238)	Patients without SSI (n=212)	Patients with SSI (n=26)	P value
**Median BMI**	29.2 (17.9-51.9)	28.5 (17.9-50.8)	36.1 (20.9-51.9)	**0.003**
**Diabetes Mellitus**				0.700
Yes	40 (16.5%)	35 (16.2%)	5 (19.2%)	
No	202(79.9%)	181 (83.8%)	21 (80.8%)	
**Smoking**				**0.020**
Yes	11 (4.5%)	7 (3.2%)	4 (15.4%)	

No	231 (95.5%)	209 (96.8%)	22 (84.6%)	
**Route of surgery**				**<0.001**
Laparotomy	94 (38.8%)	72(33.3%)	22 (84.6%)	
Laparoscopy	148 (61.2%)	144 (66.7%)	4 (15.4%)	
**Stage of disease**				**0.026**
Stage I-II	159 (65.7%)	147 (92.5%)	12 (7.5%)	
Stage III-IV	83 (34.3%)	69 (83.1%)	14 (16.9%)	
**Median Estimated blood loss (ml)**	200.0 (50 – 5500)	150.0 (50 - 5500)	325 (50-3000)	**0.010**
**ASA**	3 (1-5)	3 (1-4)	3 (2-5)	0.378
**Bowel resection**				0.131
Yes	6 (2.5%)	4 (1.9%)	2 (7.7%)	
No	236 (97.5%)	212 (98.1%)	24 (92.3%)	
**Intra-operative complications**				**0.006**
Yes	13 (5.4%)	8 (3.7%)	5 (19.2%)	
No	229 (94.6%)	208 (96.3%)	21 (80.8%)	
**Median Length of hospital stay (days)**	1.0	1.0 (0-17)	4.0 (0-101)	**<0.001**
**Re-surgery < 30 days**				0.047
Yes	4 (1.7%)	2 (0.9%)	2 (7.7%)	
No	238 (98.3%)	214 (99.1%)	24 (92.3%)	
**Re-admission < 30 days**				**0.002**
Yes	11 (4.5%)	6 (2.8%)	5 (19.2%)	
No	231 (95.5%)	210 (97.2%)	21 (80.8%)	

Table S3. Multivariable logistic regression for risk factors for SSI in patients with uterine cancer

	**p-value**	**Odds Ratio**	**95% CI**
BMI <30 kg/m2 vs >30/kg/m2	0.038	2.987	1.063 - 8.390
Smoking	0.018	7.462	1.413- 39.394
Route of surgery (laparocopy vs laparotomy)	<0.001	0.128	0.040 - 0.414
Early versus Advanced stage of disease	0.271	1.703	0.660 - 4.399

Table S4. Univariate analysis for SSI in patients with ovarian cancer

Univariate analysis of risk factors for SSI in patients with ovarian cancer				
	**Total (n=133)**	**Patients without SSI (n=121)**	**Patients with SSI (n=12)**	**P value**
**Median BMI**	26.1 (14.8 - 40.8)	26.0 (14.8 - 40.8)	29.1 (18.4 - 33.7)	0.741
**Diabetes Mellitus**				0.362
Yes	17(12.8%)	17 (14.0%)	0 (0%)	
No	116 (87.2%)	104 (86.0%)	12 (100%)	
**Smoking**				0.123
Yes	7 95.3%)	5 (4.1%)	2 (16.7%)	
No	126 (94.7%)	116 (95.9%)	10 (83.3%)	
**Route of surgery**				1.000
Laparotomy	122 (91.7%)	111 (91.7%)	11 (91.7%)	
Laparoscopy	11 (8.3%)	10 (8.3%)	1 (8.3%	
**Stage of disease**				0.070
Stage I-II	28 (21.1%)	28 (23.1%)	0	
Stage III-IV	105 (78.9%)	93 (69.9%)	12 (100%0)	
**Median Estimated bloodloss (ml)**	300 (50 -11000)	300 (50-11000)	1500 (50-4500)	0.102
**ASA**	3.0	3.0	3.0	0.378
**Bowel resection**				0.188
Yes	41 (30.8%)	35 (28.9%)	6 (50.0%)	
No	92 (69.2%)	86 (71.1%)	6 (50.0%)	
**Intra-operative complications**				0.188
Yes	9 (6.8%)	7 (5.8%)	2 (16.7%)	
No	124 (93.2%)	114 (94.2%)	10 (83.3%)	
**Median Length of hospital stay (days)**	4 (0-16)	3 (0-16)	5 (0-15)	**0.005**
**Re-surgery < 30 days**				**0.002**
Yes	4 (3.0%)	1 (0.8%)	3 (2.3%)	
No	129 (97.0%)	120 (99.2%)	9 (75.0%)	
**Re-admission < 30 days**				**0.002**
Yes	13 (9.8%)	8 (6.0%)	5 (41.7%)	
No	120 (90.2%)	113 (93.4%)	7 (58.3%)	

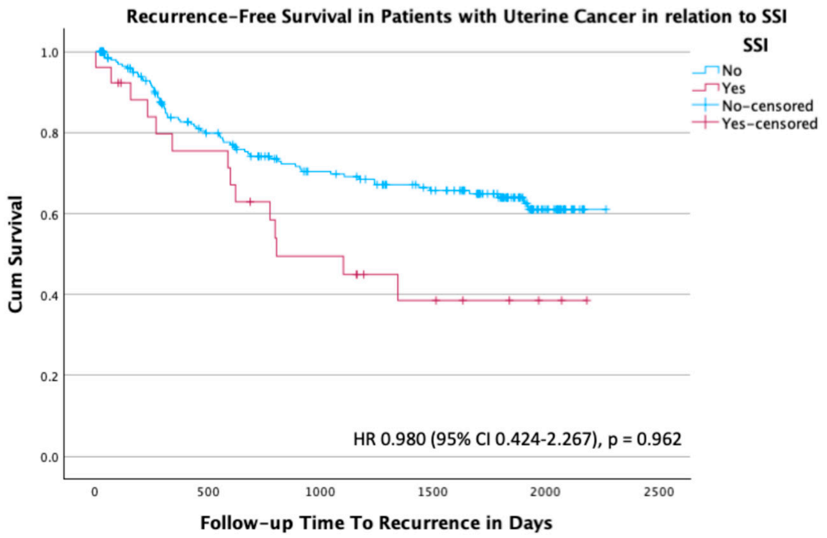

Supplementary Figure S1. Kaplan-Meier survival plot for recurrence-free survival in the subgroup of patients with ovarian cancer stratified by presence or absence of SSI, HR 0.980 (95%CI 0.424-2.67), p = 0.962).
